# Perioperative Outcomes of Primary Bariatric Surgery in North-Western Europe: a Pooled Multinational Registry Analysis

**DOI:** 10.1007/s11695-018-3408-4

**Published:** 2018-07-19

**Authors:** Youri Q. M. Poelemeijer, Ronald S. L. Liem, Villy Våge, Tom Mala, Magnus Sundbom, Johan Ottosson, Simon W. Nienhuijs

**Affiliations:** 1 Scientific Bureau, Dutch Institute for Clinical Auditing, Leiden, Netherlands; 2 Department of Surgery, Leiden University Medical Center, Albinusdreef 2, 2333 ZA Leiden, Netherlands; 3 Department of Surgery, Groene Hart Hospital, Gouda, Netherlands; 4 Dutch Obesity Clinic, The Hague, Netherlands; 5 Scandinavian Obesity Surgery Registry, Bergen, Norway; 6 Department of Gastrointestinal Surgery, Oslo University Hospital, Oslo, Norway; 7 Department of Surgical Sciences, Uppsala University, Uppsala, Sweden; 8 Department of Surgery, Örebro University Hospital, Örebro, Sweden; 9 Department of Surgery, Catharina Hospital, Eindhoven, Netherlands

**Keywords:** DATO, DICA, DSMBS, SOReg, Netherlands, Sweden, Norway, Bariatric, Surgery, Audit, Auditing, Comparison, Gastric bypass, Sleeve gastrectomy, Registry, Outcome, Quality, Indicators, Complications, Clavien-Dindo

## Abstract

**Introduction:**

The global prevalence of obesity has increased in recent decades, and bariatric surgery has become a part of the treatment algorithm of obesity. National high-quality registries enable large-scale evaluations of the use and outcome of bariatric surgery and may allow for improved knowledge. The main objective was to evaluate the rate and type of complications after primary bariatric surgery in three North-Western European countries using nationwide registries.

**Materials and Methods:**

Data from three registries for bariatric surgery were used (January 2015–December 2016). All registries have nationwide coverage with data on patient characteristics, obesity-related diseases, surgical technique, complications, grading of complications, reinterventions, readmissions, and mortality. Eligibility criteria for bariatric surgery were similar and included body mass index of ≥ 40.0 or ≥ 35.0 kg/m^2^, with one or more obesity-associated diseases.

**Results:**

A total of 35,858 procedures (32,177 primary) were registered. The most common procedure was gastric bypass in the Netherlands (78.9%) and Sweden (67.0%), and sleeve gastrectomy in Norway (58.2%). A total of 904 (2.8%) patients developed major complications after primary surgery and 12 patients (0.04%) died within 30 days. Total number of complications between the registries were comparable (*p* = 0.939). However, significant differences were seen for Clavien-Dindo Classification grades IIIb and IV (*p* < 0.001). Pooled readmission rates were 4.3% (*n* = 1386).

**Discussion:**

Bariatric surgery is safely performed in the three evaluated countries. Standardization of registries and consensus of variables are essential for international comparison and may contribute to improved quality of treatment across nations.

## Introduction

The global prevalence of obesity and associated diseases has increased considerably in recent decades. Bariatric surgery has become a part of the treatment algorithm of obesity as significant and sustained weight loss, improvements of related diseases, and health-related quality of life can be assured [1–5]. On an individual basis, the indication for surgery should be balanced against the risk for postoperative complications and side effects.

Laparoscopy has contributed to the increased use of bariatric surgery worldwide [6–8]. Perioperative mortality is generally low at 0.08–0.35%, although perioperative morbidity range from 10 to 17% [3]. A shift towards high-volume hospitals may have contributed to a reduced risk of procedure-related complications [9].

National high-quality registries enable large-scale evaluations of the use and outcome of bariatric surgery and may allow for improved knowledge. Such registries have been established in several countries. The validity of the registries relies to a large extent on the quality of data retrieved and on high coverage rates [10, 11].

The primary aim of this study was to evaluate the rate and type of complications after primary bariatric surgery in three North-Western European countries using nationwide registries. Findings could guide focus for adjustments that may improve the standard of bariatric care and may act as a benchmark analysis for comparison of outcome.

## Materials and Methods

Data from three nationwide registries for bariatric surgery were used. The Swedish registry started in 2007 as the Scandinavian Obesity Surgery Registry (SOReg) and was extended to Norway in 2014 (SOReg-N for Norway and SOReg-S for Sweden) [10]. SOReg-N received status as a nation registry in June 2015 and the two registries were coordinated to allow for common use of data. The variables registered have the same definitions and the database platform is the same. An identical system for auditing of data to improve quality has been developed in the Netherlands. The Dutch Society for Metabolic and Bariatric Surgery (DSMBS) started a mandatory nationwide clinical audit in January 2015, called the Dutch Audit for Treatment of Obesity (DATO) [11].

All three registries have a nationwide coverage and include data on patient characteristics, obesity-related diseases, surgical technique, perioperative complications, grading of the complications, reinterventions, readmissions, and mortality. Reporting to DATO is mandatory, and for this type of study, formal consent was not required under Dutch law. Reporting to SOReg-S and SOReg-N is not mandatory but “expected.” The Swedish law allows patient inclusion in SOReg-S without the need of formal consent from the patient, while for SOReg-N, a written and informed consent from the patient is obligatory according to Norwegian legislation. Each country has a validated system by an external third party providing an on-site audit on a randomly selected number of patients. All procedures performed in this study were in accordance with the ethical standards of the institutional and/or national research committee and with the 1964 Helsinki declaration and its later amendments or comparable ethical standards. Characteristics of the three registries are stated in Table 1.

**Table 1:** Characteristics of participating countries and datasets

	Netherlands	Norway	Sweden
Inhabitants (× 10^6^)	16.9	5.2	9.8
Numer of bariatric proceduresper 100,000 inhabitants	65.1	55.6	61.4
Minimum required procedures per hospital	2015: 100/year 2016: 200/year	2015: not defined 2016: not defined	2015: not defined 2016: not defined
Registry			
Registry	DATO	SOReg-N	SOReg-S
Registry active since	2015	2015	2007
Registry organization	18 hospitals 1 central database	20 hospitals 1 central database	42 hospitals 1 central database
Data availability^a^			
Patient characteristics	+	+	+
Obesity-related diseases	+	+	+
Surgical technique	+	+	+
Perioperative complications	+	+	+
Reinterventions	+	+	+
IC/ICU admission	+	+	+
Hospital stay	+	+	+
Readmission	+	+	+
Mortality	+	+	+

*DATO* Dutch Audit for Treatment of Obesity, *SOReg* Scandinavian Obesity surgery Registry, *IC* intensive care, *ICU* intensive care unit

^a^Obligatory in all registries

As DATO and SOReg-N first received nationwide status in 2015, it was chosen to compare the data from January 1, 2015 till December 31, 2016. Revisions and secondary bariatric procedures were excluded from the analysis; thus, the focus was on primary bariatric surgery. Bariatric procedures were presented in three main groups: sleeve gastrectomy, gastric bypass (including Roux-en-Y, mini/one anastomosis and banded variations), and other bariatric procedures.

Since contributing to DATO is compulsory, the estimated national coverage rate for the number of bariatric procedures performed in the Netherlands in 2015 and 2016 was 100%. Based on data from the National Patient Registries in Sweden and Norway, supplemented with data from the Norwegian Association for Bariatric Surgery, the estimated coverage rate for SOReg-S was 98% for both years while for SOReg-N, the coverage rate was 18% (531 out of 2900) for 2015 and 48% (1353 of 2846) for 2016.

Eligibility criteria for bariatric surgery were similar in the three countries. Patients with a body mass index (BMI) of ≥ 40.0 or ≥ 35.0 kg/m^2^, with one or more obesity-associated diseases were eligible for bariatric surgery [12–14]. Indication for surgery and the type of the bariatric procedure was based on the experience of the surgeon, the multidisciplinary team, and on shared decision making together with the patient. “Fast-track” principles were considered standard in the postoperative care in all three countries [15].

### Definition of Obesity Associated Diseases

Demographics and obesity-related diseases were uniformly defined and registered in the three registries. An obesity-associated disease was recorded as present if the patient reported receiving pharmacological treatment for the actual disease. Diseases recorded are type 2 diabetes mellitus (T2DM), hypertension, hyperlipidemia, gastroesophageal reflux disease (GERD), musculoskeletal pain, and obstructive sleep apnea syndrome (OSAS) with ongoing continuous or bilevel positive airway pressure (CPAP/BiPAP) treatment [2, 16–20].

Musculoskeletal pain was defined as daily use of pain-controlling medication or pain resulting in severe limitations of daily activity (e.g., unable to work) [21, 22]. This definition was fairly similar for the three registries.

### Classification of Complications

Complications within the first 30 days after surgery were registered and categorized according to the Clavien-Dindo Classification of Surgical Complications (CD) [23]. A severe complicated course is defined as CD grade IIIb or higher. A CD grade IIIb denotes a complication requiring intervention under general anesthesia, while CD grade IV was a complication requiring intensive care management and involving either single-organ dysfunction (CD grade IVa) or multiple-organ failure (CD grade IVb). Mortality is defined as CD grade V and includes death from any cause within 30 days after surgery or during the same hospital admission. Patients with multiple complications were counted only once, and the complication with the highest grade was used for analysis.

### Statistical Analysis

Univariate analysis was performed to discriminate between countries and severe 30-day complications (CD grade ≥ IIIb). Categorical variables were compared with the *χ*^2^ test with Yates’ correction, and continuous variables with a *t* test. Statistical significance was set at a threshold of 0.05.

Statistical analyses were performed with R version 3.4.2 in combination with the “Companion to Applied Regression”-package (car 2.1-5) and “A Grammar of Data Manipulation”-package (dplyr 0.7.4).

## Results

A total of 35,858 unique cases were registered during the study period (Table 2). Of these, 21,941 (61.2%) were operated in the Netherlands, 1884 (5.2%) in Norway, and 12,033 (33.6%) in Sweden. There were 3681 (10.3%) revisional procedures which were not included in subsequent analyses.

**Table 2:** Preoperative patient characteristics according to country

	Netherlands		Norway		Sweden		All		*p* value*
	*N*	%	*N*	%	*N*	%	*N*	%	
Total number of procedures	21,941		1884		12,033		35,858		–
Primary procedures	18,784	85.6%	1790	95.0%	11,603	96.4%	32,177	89.7%	< 0.001
> sleeve gastrectomy	3652	19.4%	1042	58.2%	3631	31.2%	8315	25.8%	< 0.001
> gastric bypass	14,988	79.8%	747	41.7%	7778	67.0%	23,513	73.1%	< 0.001
> other procedures	144	0.8%	1	0.1%	204	1.8%	349	1.1%	< 0.001
Revisional procedures	3157	14.4%	94	5.0%	430	3.6%	3681	10.3%	< 0.001
Patient characteristics^a^									
Age (mean, years, SD)	43.8	± 11.2	42.4	± 11.1	41.0	± 11.5	42.4	± 11.3	< 0.001
BMI (mean, kg/m^2^, SD)	43.3	± 5.4	42.7	± 5.2	41.2	± 5.7	42.4	± 5.4	< 0.001
Male/female	3863 14,921	20.6% 79.4%	417 1373	23.3% 76.7%	2652 8951	22.9% 77.1%	6932 25,245	21.5% 78.5%	< 0.001 < 0.001
Preoperative comorbidities^a^									
Type 2 diabetes mellitus	4122	21.9%	229	12.8%	1405	12.1%	5756	17.9%	< 0.001
Hypertension	6497	34.6%	523	29.2%	2849	24.6%	9869	30.7%	< 0.001
Dyslipidemia	3660	19.5%	214	12.0%	1013	8.7%	4887	15.2%	< 0.001
GERD	2078	11.1%	246	13.7%	1175	10.1%	3499	10.9%	< 0.001
OSAS	3374	18.0%	235	13.1%	1131	9.8%	4740	14.7%	< 0.001
Musculoskeletal pain	8209	43.7%	521	29.1%	2426	20.9%	11,156	34.7%	< 0.001
Other	8626	45.9%	360	20.1%	2873	24.8%	11,859	36.9%	< 0.001

*SD* standard deviation

*p values compared all three different countries together. All *p* values between the different countries were < 0.001

^a^Calculated on unique patients after primary bariatric surgery

Of the 32,177 primary interventions, 25,245 (78.5%) were performed in women. In the Netherlands, Norway, and Sweden, age and BMI distribution were fairly similar, 43.8, 42.4, and 41.0 years and 43.3, 42.7, and 41.2 kg/m^2^, respectively (Table 2). In conclusion, Dutch patients were significantly older, had a higher BMI, and had a higher number of registered obesity-related disease, compared to both Scandinavian countries.

Gastric bypass procedures were the most common procedures in the Netherlands and in Sweden (79.8 and 67.0%, respectively), while in Norway, sleeve gastrectomy was more common (58.2%, *p* < 0.001). There were significantly more preoperative obesity associated diseases registered in the Netherlands compared to Norway and Sweden (*p* < 0.001). The most frequent diseases were hypertension, T2DM, and musculoskeletal pain (Table 2).

### Complications

In 2095 patients (6.5%), a perioperative complication was noted. A total of 904 (2.8%) patients developed a major complication after primary surgery (Table 3) and 12 patients (0.04%) died within 30 days. In the pooled analysis, the most common complications after primary bariatric surgery were bleeding, leakages, and intestinal occlusion/obstruction. There was no significantly difference in the total number of complications between the registries (*p* = 0.939). However, a significant difference was seen in both CD grades IIIb and IV (*p* < 0.001) (Table 3). The Norwegian figures should be interpreted with care due to a lower coverage rate.

**Table 3:** Morbidity and mortality after primary bariatric surgery

	Netherlands		Norway		Sweden		All		*p* value*
	*N*	%	*N*	%	*N*	%	*N*	%	
Total number of procedures	18,784		1790		11,603		32,177		–
Total number of complications	1199	6.4%	162	9.1%	734	6.3%	2095	6.5%	0.939
Perioperative complications									
Gastrointestinal perforation	105	0.6%	14	0.8%	89	0.8%	208	0.6%	0.067
Bleeding	89	0.5%	N/A		18	0.2%	107	0.3%	< 0.001
Spleen injury	32	0.2%	8	0.4%	24	0.2%	64	0.2%	0.041
Hepatic injury	36	0.2%	N/A		12	0.1%	48	0.1%	0.059
Major vascular injury	2	0.0%	N/A		2	0.0%	4	0.0%	0.626
Postoperative complications									
Bleeding	263	1.4%	27	1.5%	147	1.3%	437	1.4%	0.530
Leakage	103	0.6%	20	1.1%	87	0.8%	210	0.7%	0.004
Intra-abdominal infection	26	0.1%	13	0.7%	58	0.5%	97	0.3%	< 0.001
Wound infection	26	0.1%	13	0.7%	83	0.7%	122	0.4%	< 0.001
Intestinal obstruction	46	0.2%	7	0.4%	95	0.8%	148	0.5%	< 0.001
Cardiac complications	34	0.2%	4	0.2%	9	0.1%	47	0.1%	0.049
Pulmonary complications	58	0.3%	4	0.2%	37	0.3%	99	0.3%	0.794
Thrombotic complications	5	0.0%	2	0.1%	10	0.1%	17	0.1%	0.048
Bowel injury	18	0.1%	14	0.8%	89	0.8%	121	0.4%	< 0.001
Other	356	1.9%	36	2.0%	175	1.5%	567	1.8%	0.033
Overall									
Reintervention									
CD grade IIIb	361	1.9%	41	2.3%	340	2.9%	742	2.3%	< 0.001
IC/ICU admission									
CD grade IV	128**	0.7%	4	0.2%	18	0.2%	150	0.5%	< 0.001
Mortality									
CD grade V	11	0.1%	0	0.0%	1	0.0%	12	0.0%	0.096
Length of stay and readmission									
Readmissions (< 30 days)	492	2.6%	104	5.8%	790	6.8%	1386	4.3%	< 0.001
Hospital stay (mean, days, SD)	1.7	± 3.0	1.9	± 2.1	2.1	± 4.9	–	–	< 0.001

*N/A* not available, *IC* intensive care, *ICU* intensive care unit, *CD* Clavien-Dindo Classification

*p values compared all three different countries together

^a^The DATO registry only registers ICU admission but does not distinguish whether an admission is due OSAS observations or not. Therefore, some ICU admission are not categorized as CD grade IV

## Discussion

This study showed similarities in measuring patient’s demographics, obesity-associated diseases, and perioperative outcomes, such as complications, in all three registries. The definitions of the variables also corresponded in the three compared countries.

Variation in annual hospital volumes for bariatric procedures was seen in the three analyzed European countries, with the highest volumes in the Netherlands (Table 1). Compared to the 2014 worldwide survey by the International Federation for the Surgery of Obesity and Metabolic Disorders (IFSO), the present annual number of procedures for the total population in the three studied countries (0.06%) is higher than the estimated amount for all IFSO counties as well the European region (0.02 and 0.03%, respectively), but lower than in the USA and Canada (0.08%) [24]. In the same survey, sleeve gastrectomy was found to have reached 45.9% of all procedures, followed by gastric bypass (39.6%) and adjustable gastric banding (7.4%). This contrasts with the present use of gastric bypass in the Netherlands and Sweden (79.8 and 67.0%, respectively).

The overall rate for severe postoperative complications was 2.8% (*n* = 904), which is consistent with previous studies [3, 25, 26]. The associated factors for major postoperative complications have been shown to include laparoscopic versus open surgery, older age, surgeon experience, preoperative comorbidities, and BMI [2, 13, 27, 28]. The perioperative mortality was low and well below earlier reports [27]. The mean days of postoperative hospital stay were respectively 1.7 days (NL), 1.9 days (NO), and 2.1 days (SW). Pooled 30-day readmission rates were 4.3% (*n* = 1386) (Table 3). Combined, this large series reflecting an unselected practice in the three countries underlines the safety of the bariatric programs evaluated. Our findings could be used as indicators of expected outcome of bariatric surgery in this region of Europe.

As stated in the IFSO report, close to 100% of the elective bariatric surgical procedures are performed by laparoscopy worldwide [29]. Laparoscopy has significantly reduced morbidity and mortality after bariatric surgery [6–8]. To further improve outcome, a minimum annual hospital volume of 200 bariatric procedures has been established in the Netherlands. National guidelines in Sweden recommend 100 procedures annually, but are not required, while such numbers are not applied in Norway. One of the reasons is the demographics of the compared countries. The Netherlands has a population density of 409 inhabitants per km^2^, compared to 13 per km^2^ in Norway, and 20 per km^2^ in Sweden. This could influence the number of procedures done annually in remote areas of the Nordic countries. It may also influence the readmission rate of the patients living in remote parts of the country and their access to bariatric experienced emergency facilities.

Some studies suggest an inverse relationship between surgical caseload and severe postoperative complications [3, 25, 26, 30, 31]. This relationship remains unclear, and accreditation on quality outcomes may be greater than that of volume. Experience with handling and outcome of treatment of complications may be influenced by hospital volume but also remains undefined.

To facilitate comparison of international accreditation and quality outcome data, the IFSO Global Registry was founded in 2013 [32]. The first IFSO Global Registry report in 2014 and a second report in 2016 demonstrated a widespread variation in access to surgery and baseline patient characteristics in the countries submitting data to the IFSO Global Registry [29, 32]. There are currently no standardized rules for countries participating in the registry. This results in participating countries with only one registering hospital and countries where the registry is nationally mandatory. It appears that only a selected number of hospitals in few countries, audited by independent third parties, ensure the data quality in large audits. The future may show whether an internationally organized registry offers added value over a nationwide external audited mandatory registry.

Studies on postoperative outcomes are commonly based on data from clinical trials or patient cohorts from single hospitals. Owing to selection, these series may not always reflect the daily practice in general and the external validity may be restricted. Comparing outcome across nations based on such data may thus be inappropriate [33, 34]. Nationwide clinical audits provide detailed information on patient characteristics, treatment and hospital details. This information is easily available and can be used for monitoring of quality indicators. These indicators can be used for individual hospitals to compare their performances nationally and internationally.

This article focuses on short-term complications. However, the observed differences in patient selection, type of bariatric procedure, and postoperative courses may affect the long-term outcomes. Such analysis will take place when data is available. The design of the present study entails several limitations. In merging data from two different registries (DATO and SOReg), it is important that definitions and other variables are identical. The present use of pharmacological treatment in comorbid diseases, the Clavien-Dindo classification in evaluating complications facilitates this. The overall coverage, i.e., not missing any procedures in the registry, is continuously validated against official statistics. The accuracy of entered data is checked by a special trained nurse from the SOReg head office by comparing all entries to the patients’ medical charts at regular site visits. In the Netherlands, it is done by an auditing team from the DICA [11].

The major strength of this study is the international, population-based design, the use of data from three high-quality registries including in-depth information and almost complete coverage of all patients who had bariatric surgery in the Netherlands and Sweden. Internal auditing measures are used in all three registries to improve data quality. Standardization of all registries, together with international consensus on definitions used in the registries, allow for easier comparisons between different countries and therefore international quality improvement. To our knowledge, this is the first multinational pooled registry analysis of national bariatric surgery programs in the world.

### Conclusion

Bariatric surgery is safely performed in the three evaluated countries. Standardization of registries, together with international consensus on definitions used in the registries, allow for easier comparisons between countries and therefore international quality improvement across nations.
